# Nutritional status and body composition by bioelectrical impedance vector analysis: A cross sectional study in mild cognitive impairment and Alzheimer’s disease

**DOI:** 10.1371/journal.pone.0171331

**Published:** 2017-02-10

**Authors:** Ilaria Cova, Simone Pomati, Laura Maggiore, Marica Forcella, Valentina Cucumo, Roberta Ghiretti, Giulia Grande, Fulvio Muzio, Claudio Mariani

**Affiliations:** 1 Center for Research and Treatment on Cognitive Dysfunctions, Institute of Clinical Neurology, Department of Clinical Sciences, ASST Fatebenefratelli-Sacco, “Luigi Sacco” University Hospital, Milan, Italy; 2 Dietetic and Clinical Nutrition Unit, ASST Fatebenefratelli-Sacco, "Luigi Sacco" University Hospital, Milan, Italy; University Of São Paulo, BRAZIL

## Abstract

**Aims:**

Analysis of nutritional status and body composition in Alzheimer’s disease (AD) and Mild Cognitive Impairment (MCI).

**Methods:**

A cross-sectional study was performed in a University-Hospital setting, recruiting 59 patients with AD, 34 subjects with MCI and 58 elderly healthy controls (HC). Nutritional status was assessed by anthropometric parameters (body mass index; calf, upper arm and waist circumferences), Mini Nutritional Assessment (MNA) and body composition by bioelectrical impedance vector analysis (BIVA). Variables were analyzed by analysis of variance and subjects were grouped by cognitive status and gender.

**Results:**

Sociodemographic variables did not differ among the three groups (AD, MCI and HC), except for females’ age, which was therefore used as covariate in a general linear multivariate model. MNA score was significantly lower in AD patients than in HC; MCI subjects achieved intermediate scores. AD patients (both sexes) had significantly (p<0.05) higher height-normalized impedance values and lower phase angles (body cell mass) compared with HC; a higher ratio of impedance to height was found in men with MCI with respect to HC. With BIVA method, MCI subjects showed a significant displacement on the RXc graph on the right side indicating lower soft tissues (Hotelling’s T^2^ test: men = 10.6; women = 7.9;p < 0,05) just like AD patients (Hotelling’s T^2^ test: men = 18.2; women = 16.9; p<0,001).

**Conclusion:**

Bioelectrical parameters significantly differ from MCI and AD to HC; MCI showed an intermediate pattern between AD and HC. Longitudinal studies are required to investigate if BIVA could reflect early AD-changes in body composition in subjects with MCI.

## Introduction

Due to global population aging, dementia and particularly Alzheimer's dementia (AD) are becoming a public health priority. The greatest risk factor for AD is a heterogeneous prognostic condition recognized as Mild Cognitive Impairment (MCI)[1]. Research efforts have been focusing on the identification of predictive factors of progression from MCI to AD.

A low body mass index (BMI) and weight loss concur and often precede the onset of AD [2], so they have recently been suggested as prognostic markers in MCI [3,4].

BMI is the most used epidemiological indicator of obesity, but reflects both body fat and muscle mass. Other simple anthropometric measurements are employed to measure indirectly fat mass such as waist circumference [5] or to determine muscle mass such as mid-upper arm circumference [6] and calf circumference [7]. Body composition could be easily studied by Bioelectrical Impedance Analysis (BIA), a non-invasive, low-cost, portable method based on the analysis of bioelectrical impedance in the human body at the passage of a low intensity alternating electrical current. Conventional BIA approach uses specific equations for the estimation of body composition; however assumptions of a constant hydration of the fat-free mass (FFM) could lead to substantial estimation errors when applied to individuals that differ from the sample used for validation [8]. A variant of BIA, bioelectrical impedance vector analysis (BIVA) [9] is a stand-alone procedure based on patterns of direct impedance measurements (impedance vectors). Impedance (Z vector) is a combination of Resistance (R), which expresses the opposition of intra- and extracellular body fluids to flow of an alternating current and Reactance (Xc) which consists in the capacitative component of tissues. R negatively correlates with the quantity of ionic solutions, Xc is directly related to the amount of soft tissue structures. The vectors of each subject are compared with a reference population and described in percentiles of a normal distribution of a probabilistic bivariate graph. Unlike BIA, BIVA method does not estimate any body compartment; the length and position of the Z vector supplies information about the state of hydration and body cell mass. The length of the vector indicates the level of hydration, from fluid overload (short vector, decreased resistance) to bodily dehydration (longer vector, increased resistance). Laterally vector displacements due to high or low reactance denotes an increase or a decrease of dielectric mass (membranes and tissue interfaces) of soft tissues. The phase shift of the tissue interfaces, called phase angle (PA), represents both the quantity and quality of soft tissue and can be calculated directly as arctan (Xc/R). Clinically it is the most important impedance parameter, predicting morbidity and mortality in a variety of diseases; higher values of PA correspond to a higher cellularity and a better cell membrane integrity [10]. Phase angle decreases with age and is significantly lower in women, due to the lower amount of body muscle [10].

Previous studies reported different bioelectrical variables indicating a lower body cell mass in patients with AD with respect to controls [11–13]. The aim of the present study was to analyze body composition characteristics by using BIVA in patients with AD and subjects with MCI in comparison with an elderly healthy control sample. To our knowledge this is the first application of bioelectrical impedance vector analysis in subjects with MCI.

## Materials and methods

### Subjects and design

This cross-sectional study was carried out in the Center for Research and Treatment on Cognitive Dysfunctions of the Luigi Sacco Hospital, University of Milan.

The study protocol was approved by the Sacco Hospital Ethics Committee and informed written consent from all subjects was obtained by the principal researcher, after neuropsychological assessment of the patients' capacity to provide a consent.

We enrolled outpatients consecutively admitted from December 2014 to January 2016 with diagnosis of MCI (by NIA-AA criteria [14]) and mild to moderate probable AD with increased level of certainty with a documented decline (by NIA-AA criteria [15]). Cognitively healthy controls (HC) were enrolled in the same period and consisted of patients’ spouses or relatives of Neurology department inpatients (hospitalized due to acute disease of CNS, such as stroke) and outpatients (mostly patients with Chronic Inflammatory Demyelinating Polyneuropathy); a minority of controls was represented by subjects with cognitive impairment.

Individuals were excluded if aged < 65 years, if they had pacemakers, heart defibrillators or other electrical implants and if they were suffering from a known active cancer.

All study participants underwent an evaluation following a standardized protocol. Collected data included demographic characteristics, medical history, present and previous pharmacological treatments. MCI and AD participants were also evaluated with an extensive neuropsychological assessment [3], clinical and neurological examination, standard laboratory blood tests and neuroimaging (MRI or CT scan). In our center, AD patients are usually requested to return every 6 months for clinical follow up visits to monitor cognitive status, level of functioning based on information from caregivers and subsequently adjust therapies.

HC underwent cognitive screening with Mini Mental State Examination (MMSE) [16] and mood evaluation with Geriatric Depression Scale (GDS) [17].

### Nutritional evaluation

Nutritional evaluation was performed by means of anthropometry, Mini Nutritional Assessment (MNA) and bioelectrical impedance vector analysis.

Anthropometric measurements were taken by the principal researcher following standard criteria [18]. Height (cm) was measured with an anthropometer and weight (Kg) with a mechanical beam scale; body mass index (BMI) (kg/m2) was hence calculated. Body circumferences (waist, mid arm and calf) were obtained with an inelastic plastic-fiber tape measure (to the nearest 1 cm); the waist was measured midpoint between the lowest rib and the upper border of the iliac crest; the mid arm was measured at the midpoint between the lateral tip of the acromion and the most distal point on the olecranon; the calf was measured at the maximum girth [6].

MNA [19] is an 18-item tool used to assess nutritional risk in elderly, grouped in 4 rubrics: anthropometric assessment (BMI, weight loss, arm and calf circumferences; items B, F, Q and R); general assessment (lifestyle, medication, mobility and presence of signs of depression or dementia; items C, D, E, G, H and I); short dietary assessment (number of meals, food and fluid intake, and autonomy of feeding; items A, J, K, L, M and N); and subjective assessment (self perception of health and nutrition; items O and P). Each answer has a numerical value and contributes to the final score, which has a maximum of 30; AD patients were helped to complete it by their caregivers. MNA screening (sum of items from A to F), global (sum of items from G to R) and total score were collected.

Bioimpedance measurements were carried out by a trained investigator (IC) in subjects fasted for at least three hours. The bioelectrical variables of resistance (R, Ohm) and reactance (Xc, Ohm) were measured with a single frequency impedance analyzer (EFG-ElectroFluidGraph; AKERN-Srl, Florence, Italy). The accuracy was checked with a calibration circuit of known impedance (R: 383 Ohm, Xc: 45 Ohm, 1% error). Whole body impedance measurements were taken using the standard positions of outer and inner electrodes on the right hand and foot [20]. The length of the impedance vector (Z) was calculated by the equation Z = (R^2^+Xc^2^)^0,5^ and the phase angle (PA) by arctan (Xc/R). The R, Xc and Z values were divided by the subject’s height (H) to remove the effect of conductor length [9].

Impedance measurements standardized by height were represented as bivariate vectors with their confidence intervals, which are ellipses in the R-Xc plane.

### Covariates

Potential confounders included personal data, such as age, sex, years of education, MMSE score, GDS score, smoking habits (previous smoker, actual smoker, no smoker). Somatic comorbidity was quantified using the Modified Cumulative Illness Rating Scale (CIRS) [21]. The modified CIRS includes 14 categories assessing the impairment of each organ system, with a score ranging from 0 to 4. The total score was calculated adding the scores from each of the 14 individual system scores. The “CIRS comorbidity index”, based on the sum of CIRS items with scores ≥ 2 (indicating moderate disability or morbidity and/or requirement of first line therapy) was also calculated. We evaluated comorbidity with particular emphasis on disease and treatments which could play a role in body composition, such as diabetes mellitus, dysthyroidism (hypothyroidism or hyperthyroidism), depression (clinical depression with/without treatment and/orGDS score ≥ 11), use of antihypertensives, especially diuretics, oral hypoglycaemic agents, insulin, levothyroxine and antidepressants.

### Statistical analysis

Subjects’ characteristics among the three groups of participants (HC, MCI and AD subjects) were compared separately for males and females as previously proposed [12, 22] using univariate ANOVA test for continuous variables and Pearson’s χ^2^ test for categorical variables. Post hoc analyses with Bonferroni correction were performed when appropriate. Multivariate analyses with general linear models were then carried out, with nutritional indicators, anthropometric and bioelectrical variables as the dependent variable, the dementia diagnostic group (normal, MCI, and AD) as the independent variable and other significant demographic and psycho-functional variables emerged in previous univariate analyses as potential confounding variables as covariates. Three general linear models were carried out: unadjusted, partially adjusted (for sociodemographic variables such as age, gender and education) and fully adjusted models (for sociodemographic variables and psycho-functional status).

The differences between the mean impedance vectors in AD, MCI and HC groups were assessed with Hotelling’s T^2^ test, a multivariate extension of the univariate t-test and graphically with 95% probability confidence ellipses. Separate 95% confidence ellipses correspond to statistically significant difference between mean vector displacements on the R-Xc plane (P < 0.05, which corresponds to a significant Hotelling’s T^2^ test, which is equivalent to a significant difference in R, Xc or both parameters).

Mahalanobis D distance (D) among mean vectors, which uses within-groups variation (elliptical shape) as a yardstick for differences between means, was also calculated.

All p values *<*0.05 were regarded as statistically significant. SPSS for Windows (version 23.0) was used for statistical analyses. BIVA was performed with an open source specific software [23].

## Results

Tables 1 and 2 show psycho-functional, anthropometric, multidimensional and bioelectrical variables in healthy controls (HC), Mild Cognitive Impairment (MCI) and Alzheimer’s disease (AD), respectively in men and in women.

**Table 1:** Descriptive and comparative statistics for the psycho-functional, anthropometric, multidimensional and bioelectrical variables in healthy controls (HC), Mild Cognitive Impairment (MCI) and Alzheimer’s disease (AD) men.

	HC (N = 29)	MCI (N = 14)	AD (N = 24)	F	Post Hoc §
**Demographic variables**					
Age (y)	74.3 ± 5.3	78.6 ± 4.4	77.5 ± 8.2	2.85	
Education (y)	10.2 ± 3.9	10.1 ± 4.8	9.7 ± 3.8	0.11	
**Psycho-functional indicators**					
MMSE score	29.2 ± 0.9	25.1 ± 2.5	19.7 ± 4.4	68.78***	AD<MCI <HC
GDS score^a^	6.6 ± 3.6	5.1 ± 3.6	11.8 ± 5.1	7.34***	HC = MCI<AD
ADL (functions lost)	0.0 ± 0.0	0 ± 0	1.6 ± 1.8	16.73***	AD<MCI = HC
IADL (functions lost)	0.0 ± 0.0	0.7 ± 0.1	3.5 ± 1.7	159.29***	AD<MCI = HC
**Nutritional indicators**					
MNA screening score	13.9 ± 0.4	12.9 ± 1.3	11.9 ±1.2	24.78***	AD< MCI<HC
MNA global score	14.2 ±1.1	14.2 ± 1.3	12.2 ± 1.4	19.06***	AD<MCI = HC
MNA total score	28.0 ± 1.1	27.1 ± 1.9	24.1 ± 2.3	33.04***	AD<MCI = HC
**Anthropometric variables**					
BMI (kg/m^2^)	26.6 ± 2.5	26.5 ± 3.0	24.8 ± 3.0	3.12	
Arm circumfence (cm)	26.9 ± 2.8	26.1 ±2.0	24.0 ± 3.0	7.96**	AD<HC
Calf circumfence (cm)	35.2 ± 2.8	33.6 ± 3.1	32.9 ± 2.3	4.86*	AD<HC
Waist circumfence (cm)	96.7 ± 7.9	99.1 ± 9.4	90.4 ±10.6	4.86*	AD<MCI
**Bioelectrical variables**					
Rz/h (Ώ/m)	231.8 ± 23.8	261.0 ± 34.2	260.6 ± 35.4	7.29**	HC<MCI = AD
Xc/h (Ώ/m)	26.7 ± 3.2	29.5 ± 6.5	27.2 ± 4.8	1.77	
PA (°)	6.6 ± 0.7	6.4 ± 0.7	5.9 ± 0.6	6.39**	AD<HC
Z/h (Ώ/m)	233.3 ± 23.9	262.0 ± 34.6	262.1 ± 35.6	7.20**	HC<MCI = AD

values are expressed as mean ± standard deviation.

^§^ only significant differences are shown.

^a^ available for HC, MCI and 28% AD group.

* p<0.05.

** p<0.01.

*** p< 0.001.

**Table 2:** Descriptive and comparative statistics for the psycho-functional, anthropometric, multidimensional and bioelectrical variables in healthy controls (HC), Mild Cognitive Impairment (MCI) and Alzheimer’s disease (AD) women.

	HC (N = 29).	MCI (N = 20).	AD (N = 35)	F	Post Hoc §	F ^♯^
**Demographic variables**						
Age (y)	75.1 ± 6.4	76.9 ± 4.5	82.1 ± 4.8	14.82***	HC<AD; MCI<AD	
Education (y)	8.0 ± 3.4	6.5 ± 3.5	6.1 ± 3.3	2.46		3,81*
**Psycho-functional indicators**						
MMSE score	29.2 ± 0.9	25.9 ± 2.5	18.9 ± 4.9	75.42***	AD<MCI; MCI<HC	50,12***
GDS score^a^	7.6 ± 4.0	10.7 ± 5.6	7.6 ± 5.0	2.56		1,96
ADL (functions lost)	0.0 ± 0.0	0.0 ± 0.0	1.9 ± 1.8	27.09***	HC = MCI<AD	21,87***
IADL (functions lost)	0.0 ± 0.0	0.3 ± 0.6	5.0 ± 2.0	104.93***	HC<MCI<AD	102,38***
**Nutritional indicators**						
MNA screening score	12.7 ± 1.4	12.4 ± 2.4	11.8 ± 1.4	2.24		2,02
MNA global score	14.4 ± 1.1	13.0 ± 1.4	11.6 ± 1.7	28.80***	AD<MCI <HC	19,90***
MNA total score	27.1 ± 4.5	25.4 ± 3.1	23.4 ± 2.7	16.62***	AD<HC	12,11***
**Anthropometric variables**						
BMI (kg/m^2^)	27.2 ± 4.5	25.8 ± 4.3	25.1 ± 3.9	1.87		1,88
Arm circumfence (cm)	26.9 ± 3.2	25.4 ± 2.8	25.0 ± 3.1	3.02		3,07*
Calf circumfence (cm)	33.9 ± 2.8	33.1 ± 2.7	30.9 ± 3.5	7.68**	AD<HC = MCI	5,58**
Waist circumfence (cm)	93.0 ± 12.2	86.4 ± 9.5	86.4 ± 10.5	3.48		2,37
**Bioelectrical variables**						
Rz/h (Ώ/m)	286.8 ± 34.3	303 ± 38.2	311.9 ± 30.9	4.40*	HC<AD	3,30*
Xc/h (Ώ/m)	30.2 ± 4.1	29.3 ± 3.4	29.5 ± 5.0	0.30		4,71**
PA (°)	6.1 ± 0.6	5.6 ± 0.6	5.4 ± 0.7	6.39*	AD<HC	17,54***
Z/h (Ώ/m)	288.4 ± 34.4	305 ± 38.2	313.4 ± 31.0	7.20*	HC<AD	3,21*

Values are expressed as mean ± standard deviation.

^§^ only significant differences are shown.

^a^ available for HC, MCI and 14,3% AD group.

* p<0.05.

** p<0.01.

*** p< 0.001.

^♯^ general linear multivariate model (adjusted for age).

Sociodemographic variables did not differ among AD, MCI and HC, except for females’ age (HC <AD; MCI <AD), whereby age was then used as covariate in a general linear multivariate model (Table 2). AD patients were enrolled after 68.1 ± 12 months and MCI after 64.1 ± 43.5 months from the onset of cognitive impairment.

AD, MCI and HC were similar in terms of factors potentially confounding the relationship between body composition and dementia process (smoke habit, diabetes mellitus, use of oral hypoglycemic agents/insulin, dysthyroidism, use of levothyroxine, use of diuretics/other antihypertensives, use of antidepressants), with the exception of clinical depression which was more prevalent among women with MCI than AD and HC (Pearson's χ^2^ test p<0.05; post hoc: HC<MCI; AD<MCI) and CIRS total score which was higher in MCI with respect to AD and HC (0.5 ± 0.2 vs. 0.3± 0.2 vs. 0.4 ±0.2, p = 0.003; post hoc: HC<MCI; AD<MCI).

MNA global and total score were lower in AD than in HC in both sexes; MCI did not differ from HC except for a lower MNA screening score in men group and for a lower global score in women group.

Interestingly, when analysing each MNA subitems, hydration (item M) results significantly reduced in AD patients with respect to MCI and HC (Pearson’s χ^2^ test; men p<0.05, women p<0.001).

With regards to anthropometric measurements, AD of both sexes showed significantly lower arm and calf circumferences with respect to HC (women’s arm circumferences in general linear multivariate model: F 3.07, p <0.05; AD< MCI = HC). AD men had lower waist circumferences than HC and MCI.

The phase angle (PA), ratio of reactance to height (R/h) and ratio of impedance to height (Z/h) were significantly different in AD patients from HC in both sexes; women with AD showed also a significantly lower ratio of reactance to height (Xc/h) than HC in the general linear multivariate model with age used as a covariate (F 4.71, p <0.01). A higher ratio of reactance to height (R/h) and ratio of impedance to height (Z/h) was found in men with MCI with respect to HC.

No statistically significant differences in bioelectrical parameters (PA, R/h, Xc/h, Z/h) have been found between women with MCI with and without depression.

Unadjusted, partially adjusted and fully adjusted general linear models showed overlapping results.

Given the small study sample, a sensitivity analysis with non parametric tests (Kruskal Wallis) was also performed and confirmed the results.

No significant correlation emerged between disease duration (from disease onset) and nutritional assessment with MNA or bioelectrical parameters.

**Fig 1:**
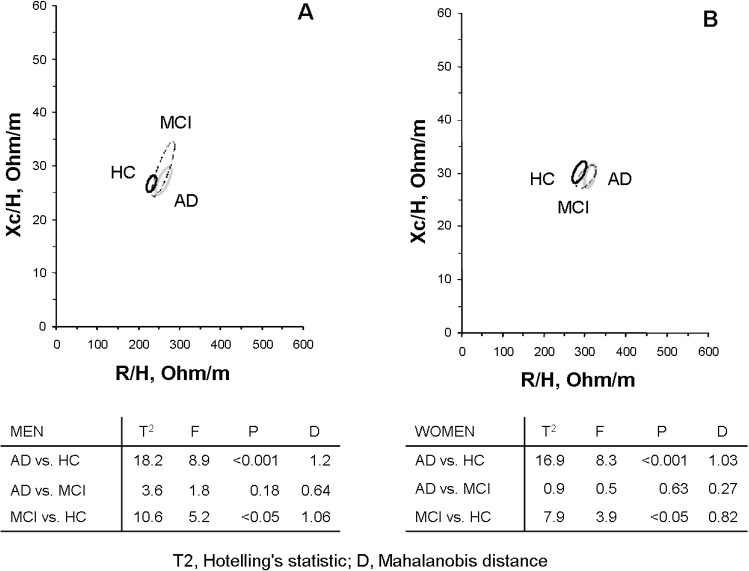
**A** Distribution of confidence ellipses of men with Alzheimer’s dementia (AD), Mild Cognitive Impairment (MCI) and healthy controls (HC). **B** Distribution of confidence ellipses of women with Alzheimer’s dementia (AD), Mild Cognitive Impairment (MCI) and healthy controls (HC).

Mean impedance vector and confidence ellipses are shown in Fig 1A and 1B; statistical comparison of groups with Hotelling's T^2^ test, with the corresponding p value and Mahalanobis distance D are also reported.

The 95% confidence ellipses significantly differed between HC and AD (Hotelling’s T^2^ test: men = 18.2; women = 16.9; p < 0.001). The ellipses of the patients with AD shifted toward the inferior region of the RXc graph, corresponding to low body cell mass (Fig 1). The ellipses of subjects with MCI were closer to AD ellipses than HC, and significantly differed from HC (Hotelling’s T^2^ test: men = 10.6; women = 7.9; p < 0.05).

## Discussion

According to our results, patients with AD showed a significantly different nutritional status with respect to cognitively healthy controls. Anthropometric measurements, more precisely calf and arm circumferences, were significantly lower in AD patients in both sexes; waist circumferences resulted significantly lower in men with AD than in controls. Patients with AD (both sexes) achieved lower score at MNA. Worse nutritional indicators at BIVA were found in AD patients with respect to controls: bioelectrical vectors were characterized by lower phase angles, higher height-normalized impedance values and a different pattern of impedance vector migration with displacement on the right side of the confidence ellipses of AD patients with respect of controls. MCI subjects did not differ from controls in anthropometric measures and in nutritional assessment by MNA except for screening score in men group. According to BIVA, men with MCI showed higher impedance vectors (normalized for height) and significantly different patterns of impedance vector migration were found in MCI of both sexes with respect to controls.

While our results in AD patients are consistent with available literature, to our knowledge this is the first study analysing body composition by BIVA in subjects with MCI. In 2010 Buffa et al. [12] first applied BIVA in AD patients and found significantly lower phase angles (indicating lower soft tissue) in patients with mild-moderate AD of both sexes with respect to controls; furthermore they found that women with severe AD showed reduced tissue mass and dehydration when compared with AD patients with mild-moderate disease severity. In 2012 the same group [22] detected higher impedance values (Z/h and R/H) in AD patients than in controls and suggested an increase of fat component with respect to the muscle mass along with psycho-functional decline: this hypothesis was supported by replication of these findings with the technique of specific BIVA[11].

We have noticed that nutritional status of our control and AD population completely differs from Sardinian population enrolled in the study of Saragat et colleagues [11,22], since we detected significantly lower impedance values (R/H, Xc/H and Z/h) in both populations. This finding underlines the importance of recruiting a local control population in nutritional studies, because differences in body composition and hydration status even among regions of the same country can be conspicuous.

Recently, it has been suggested that BIVA could reflect dementia-related changes in body composition better than BIA in a study which involved men with dementia (undefined subtype of dementia) [13]. This could be mainly due to the fact that fat free mass has not the same hydration percentage (73%) as conventional BIA presupposes. This is the reason why we preferred to avoid calculating fat mass (FM) and fat free mass (FFM) in the present study. Our findings of this particular alteration of electrical properties of tissues in AD patients support the hypothesis of lower muscle mass and consequently higher fat mass. During aging process, reduction of body weight, height and FFM, associated with an increase in FM is well documented [24]. However, body composition of elderly subjects with AD differs from that of cognitively healthy elderly subjects [25]: lower arm and calf circumferences and bioelectrical differences in AD patients with respect to controls in the present study corroborate this hypothesis. Right side displacement of impedance vector in AD group indicates lower values of body cell mass [9] and therefore worse nutritional parameters.

MCI could be a preclinical phase of AD or other type of dementia since it has a progression rate around 10–15% per year in memory clinics [26]. Our previous works showed that a low BMI could predict progression of MCI, as well as weight loss, and several biologically plausible hypothesis have been previously proposed [3,4]. In this cross-sectional study we found significantly different impedance parameters in men with MCI; both sexes had significantly different confidence ellipses in RXc graph with respect to controls, meaning decreased conductive tissue mass (sarcopenia); this suggests that soft tissue mass could decrease with cognitive impairment independently from aging process.

Analysis of body composition with BIVA could then detect early changes in body composition which could reflect early systemic manifestation of the AD process [27]; longitudinal studies will be required to understand if a BIVA pattern indicating a worse nutritional status could be an early and sensitive marker of progression to dementia or specifically to AD in MCI subjects.

In our study, MCI subjects showed a higher comorbidities total score assessed by CIRS with respect to healthy controls and AD. Subjects who suffer from several diseases may have worse performances on cognitive assessments; mild cognitive impairment at baseline in subjects with multimorbidities may have less probability to progress in a major cognitive disorder during follow up [28]. Nevertheless, our sample of MCI did not differ from HC and AD in diseases and/or treatments potentially confounding the relationship between body composition and dementia process, such as endocrinopathies (diabetes mellitus and dysthyroidism) and cancer (since neoplastic patients have not been included in the present study); a significant difference has instead been found in clinical depression between women with MCI with respect to HC and AD. A depressed mood in women with MCI could be explained by possible self-awareness of cognitive impairment [29]. However, sensitivity analysis showed that no differences accounted for bioelectrical parameters of BIVA in depressed and not depressed women with MCI. No statistical differences has been found in depression between AD, MCI and HC men; higher GDS scores in AD patients with respect to MCI could be possibly attributable to a reduced statistical power of the analysis (GDS scores were available for 28% of men with AD).

Our results should be interpreted within the context of the limitations of the study. First, the sample size was small; therefore, further studies with a larger sample size are required in order to confirm our data. Second, since this was a cross-sectional study, we cannot determine the direction of causality between the nutritional status and cognitive outcome. Eventually, our study lacks of biomarkers’ investigation; however, the subjects classified as MCI respected international core clinical criteria of 2011 [14].

The strengths of our study are the clinical setting where the study took place, which allows to well characterize from a psychometric point of view subjects with MCI and AD; furthermore this setting facilitated detailed baseline collection of several potential confounding variables (comorbidities–particularly with regard to metabolic disorders and depression–and treatments). Finally participants of this study are representative of those who routinely consult memory clinics.

Increasing the cohort of MCI subjects and longitudinal observation will provide further information to allow generalization to populations of MCI attending memory clinics. Further studies will be also needed to evaluate nutritional status and bioimpedance analysis patterns in other types of dementia than Alzheimer’s. Considering the differences found in MNA scores among different cognitive groups, we suggest to implement clinical practice of cognitively impaired patients with this simple questionnaire, also to address nutritional advice when malnutrition is suspected.

In summary, since little is known about nutritional status of MCI subjects, our work contributes to the growing research interest in this area. In any cross-sectional study, we cannot discriminate the direction of causality. Our finding should be considered tentative until future studies confirm or disprove our observations.
